# Spectral Zones-Based SHAP/LIME: Enhancing Interpretability in Spectral Deep Learning Models Through Grouped Feature Analysis

**DOI:** 10.1021/acs.analchem.4c02329

**Published:** 2024-09-18

**Authors:** Jhonatan Contreras, Andreea Winterfeld, Juergen Popp, Thomas Bocklitz

**Affiliations:** † Institute of Physical Chemistry (IPC) and Abbe Center of Photonics (ACP), Member of the Leibniz Centre for Photonics in Infection Research (LPI), Friedrich Schiller University Jena, Helmholtzweg 4, 07743 Jena, Germany; ‡ Leibniz Institute of Photonic Technology, Member of the Leibniz Centre for Photonics in Infection Research (LPI), Member of Leibniz Health Technologies, Albert Einstein Straße 9, 07745 Jena, Germany

## Abstract

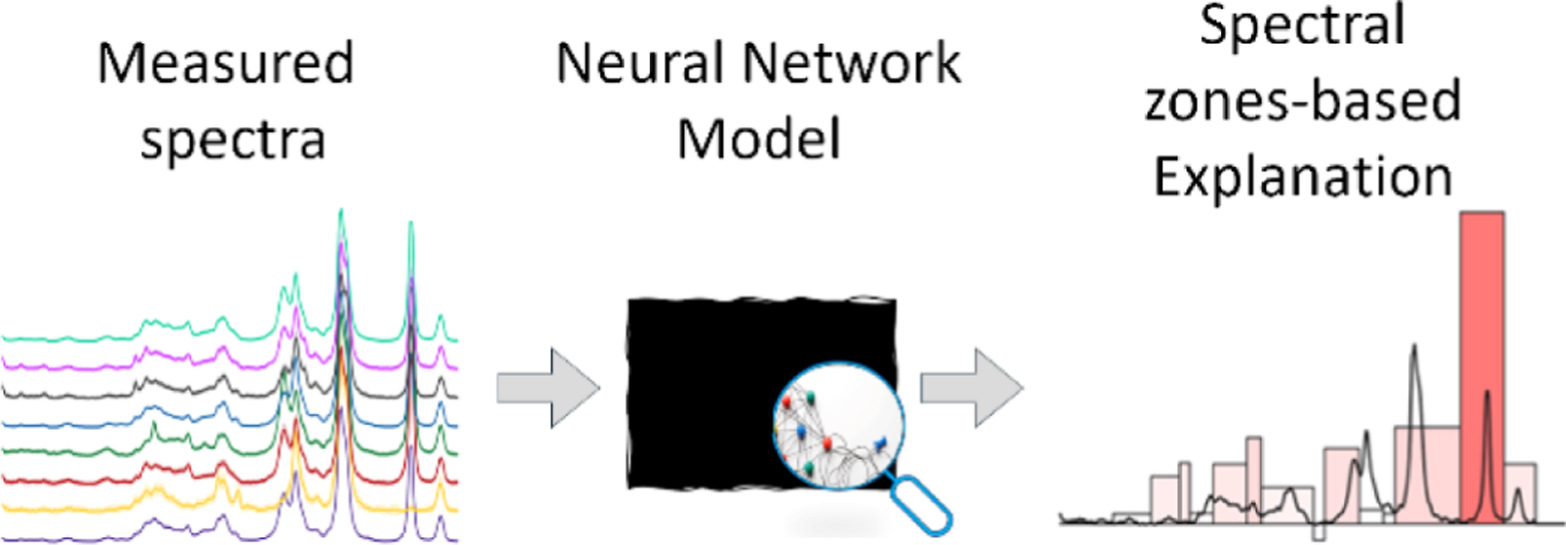

Interpretability is just as important as accuracy when it comes to complex models, especially in the context of deep learning models. Explainable artificial intelligence (XAI) approaches have been developed to address this problem. The literature on XAI for spectroscopy mainly emphasizes independent feature analysis with limited application of zone analysis. Individual feature analysis methods, such as shapley additive explanations (SHAP) and local interpretable model-agnostic explanations (LIME), have limitations due to their dependence on perturbations. These methods measure how AI models respond to sudden changes in the individual feature values. While they can help identify the most impactful features, the abrupt shifts introduced by replacing these values with zero or the expected ones may not accurately represent real-world scenarios. This can lead to mathematical and computational interpretations that are neither physically realistic nor intuitive to humans. Our proposed method does not rely on individual disturbances. Instead, it targets “spectral zones” to directly estimate the effect of group disturbances on a trained model. Consequently, factors such as sample size, hyperparameter selection, and other training-related considerations are not the primary focus of the XAI methods. To achieve this, we have developed a modified version of LIME and SHAP capable of performing group perturbations, enhancing explainability and realism while minimizing noise in the plots used for interpretability. Additionally, we employed an efficient approach to calculate spectral zones for complex spectra with indistinct spectral boundaries. Users can also define the zones themselves using their domain-specific knowledge.

## 1 Introduction

Artificial intelligence (AI) has been used in numerous computer science sectors in recent years, with notable applications in medicine and chemical analysis. Raman spectroscopy is an example of this integration.^1^ This technique facilitates the acquisition of a substance’s unique “vibrational fingerprint” through its distinct Raman spectrum, providing valuable insights into molecular vibrational states. Raman spectroscopy is a nondestructive, noninvasive provision of molecular information, and it has significantly impacted extensive applications ranging from material identification, disease diagnosis,^2,3^ environmental monitoring,^4,5^ and the food industry.^6^

Spectral data interpretation typically involves chemometric algorithms, including partial least squares regression,^7^ random forest,^8^ support vector regression, linear regression, and support vector machine.^9^ More recently, deep learning algorithms^10−12^ have shown that they outperform classical methods markedly advanced data modeling, classification, and regression tasks within spectral analysis. Unlike traditional methods, deep learning automatically extracts relevant features, reducing the need for manual preprocessing and feature selection. It captures complex nonlinear relationships, generalizes to new, unseen data, and enhances real-world applicability. Additionally, deep learning frameworks can integrate other data types (e.g., images, metadata), enhancing spectral analysis’s accuracy and robustness when applied to larger data sets.^13,14^

However, AI models frequently suffer from a lack of transparency and explainability, which hinders their acceptance among healthcare professionals and chemical researchers. In response, explainable artificial intelligence (XAI) has become a focal point of contemporary research, aiming to deliver clear, understandable explanations for AI model predictions and functions.^15^ Nevertheless, the application of XAI in spectroscopy remains relatively unexplored. Few studies have been conducted, and there is a clear trend of adapting methods from other data types, such as images and multivariate data, to spectroscopic analysis. This presents a distinct set of challenges for data interpretation within machine learning algorithms for spectral analysis. Notably, among the various XAI techniques utilized in these studies, shapley additive explanations (SHAP),^16−19^ local interpretable model-agnostic explanations (LIME),^20,21^ class activation mapping,^22,23^ Gradient-based methods,^24,25^ and global surrogate^26,27^ are the most prevalent.

Perturbation-based models such as LIME and SHAP quantify the reactions of AI models to sudden modifications in individual feature values and use them to identify the most influential features. However, sudden changes in spectra, such as replacing intensity values at some wavenumber with zero values, the mean spectra, or a value in a reference data set at that location, may not accurately reflect real-world conditions. This can lead to computational and mathematical interpretations that, while theoretically plausible, may not align with physical reality or human intuition. Alternatively, we propose to modify not individual features but spectral zones, thereby estimating the effect of the joint perturbations. This approach visualizes trained models directly, making issues such as sample size, hyperparameter selection, and other training-related considerations less critical. Consequently, we present a variant of the LIME and SHAP approaches that account for group perturbations.

Our approach involves three stages. It starts by determining spectral zones, which can be established through data-driven approaches using either a single spectrum or a data set for local or global understanding. Different methods can be employed, for instance, identifying local maxima and minima to delineate peaks and valleys in the spectra or user-defined delineations based on expert knowledge. In the second stage, representative perturbations are defined. It is crucial to create such perturbations. If the existing data set adequately represents the spectrum’s feature space, it can be directly used to substitute spectral zones. However, if the data set is inadequate, the spectral zones are augmented employing sharpening or smoothing to introduce variability. In cases where a data set is lacking, we augment the input spectra to create the necessary spectral diversities. The final stage involves computing SHAP or LIME values using the spectral zones and generating visualization plots that help understand how spectral features influence the model’s predictions.

## 2 Materials and Methods

In the domain of spectroscopy-based explainable AI, the most widely used methods involve perturbing the input data and analyzing variations in the model output to determine the importance of different features and potential bias. Popular techniques LIME and SHAP rely on this approach.

### 2.1 Local Interpretable Model-Agnostic Explanations

LIME denotes the original prediction model to be explained as *f* and explains it as a model *g* that belongs to a set of potentially interpretable models *G*, such as linear models, which are inherently interpretable with an explainable complexity Ω(*g*). The aim is to find an explanation that minimizes the loss function  in the vicinity of an interpretable instance *x* represented as π_*x*_, while keeping the complexity of the explanation low, represented as
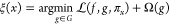


LIME focuses on the local explanation of the prediction *f*(*x*) for the single input *x*. Here, perturbed subset *z* from the perturbation set *Z* are used to approximate the loss function  employing sparse linear models. In this context, π_*x*_(*z*) denotes the measure of distance between *x* and *z* in the following equation
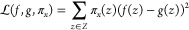


### 2.2 Shapley Additive Explanations

SHAP employs the coalitional game theory to provide explanations based on the contributions of individual feature values. The classic approach requires retraining the model on all feature subsets *S* ⊆ *F*, with *F* denoting the complete set of all features of an input *x*. Each feature is assigned an importance value that illustrates its impact on the model prediction when it is included in a subset.

In neural networks, it is impossible to exclude features. Instead, we refer exclude to “replace” by zero, the expected mean value, or a random value among the possible values, for instance, a value from the training set. Accordingly, the computation of an importance value involves assessing the model’s outcome, denoted as *f*_*S*∪{*i*}_, incorporating a particular feature alongside the model’s outcome *f*_*S*_, where the same feature is excluded. These outcomes are then compared against the input *x*_*S*_, which represents the values of the input features in the set *S*. Subsequently, Shapley values are computed and employed as feature attributions. These values are essentially a weighted average of all possible subsets *S* ⊆ *F*{*i*}, expressed as
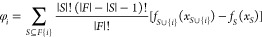


### 2.3 Limitations of LIME and SHAP

LIME and SHAP applications have limitations that must be considered. First, the computational complexity of calculating Shapley values presents a significant challenge as it scales exponentially as O (2^*F*^) with the number of features (*F*). As *F* increases, the implementation is computationally infeasible, as in spectroscopy, where the data consist of several hundreds of intensity values. Two approximation methods can be used: Deep SHAP and Sampling SHAP Explainer. For Deep SHAP Explainer, backpropagation inspired by the gradient-based approach by Ancona et al.^28^ is used in its implementation. On the other hand, Sampling SHAP Explainer involves approximating the formula by evaluating it across *N* randomly selected subsets. This reliance on approximations can lead to varying outcomes, compromising the reliability and consistency of the interpretations.

**Figure 1:**
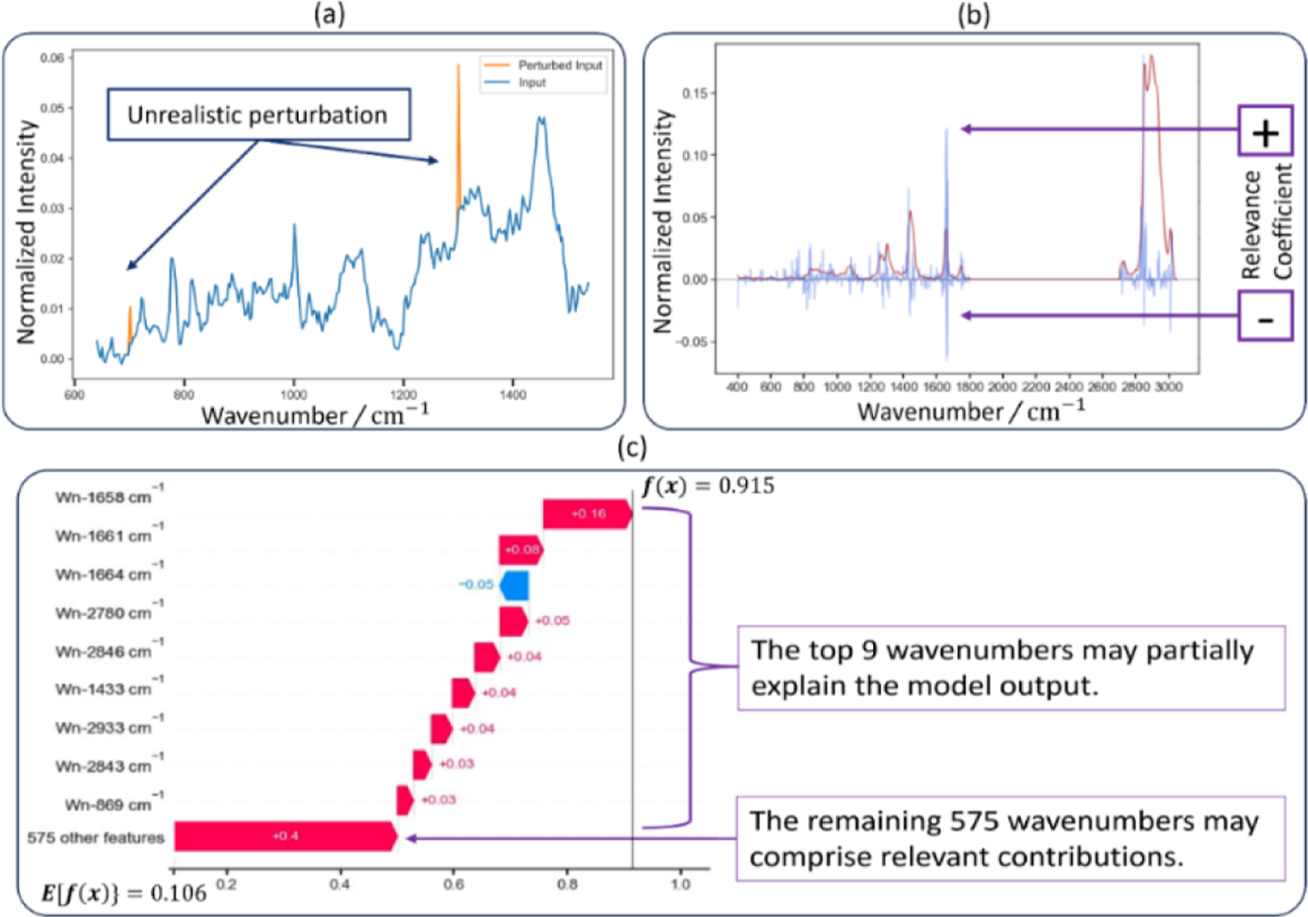
Limitations of SHAP. (a) Perturbations may not reflect real conditions. (b) Implausible interpretations with contradictory contributions at adjacent wavenumbers. (c) Top wavenumbers only partially explain the model output.

Second, the LIME and SHAP methodologies rely on individual feature perturbations and may not align well with real-world conditions. Such perturbations could lead to interpretations that do not accurately reflect practical scenarios, as shown in Figure 1a, disconnecting theoretical model understanding from practical application.

Third, LIME and SHAP may produce implausible interpretations, particularly when dealing with features that have closely related values or effects. This issue is apparent in cases like spectral analysis, where a XAI approach might assign positive and negative contributions to neighboring wavenumbers, resulting in interpretations lacking real-world relevance as shown in Figure 1b.

Finally, interpretability plots such as relevance coefficients, waterfall plots, beeswarm, force plots, and saliency maps can be difficult to interpret due to the high variance of the relevance coefficients or SHAP values in feature contribution approaches. Although waterfall plots can be helpful, they do have certain limitations. Waterfall plots that focus only on the top 9 wavenumbers in spectral analysis may result in an incomplete understanding of the model behavior, as the remaining features may carry a significant portion of the feature importance, as illustrated in Figure 1c.

### 2.4 Spectral Zones-Based SHAP/LIME

Our methodology for spectral analysis involves three steps: defining spectral zones, conducting group perturbations, and computing coefficients.

#### 2.4.1 Spectral Zones

**Figure 2:**
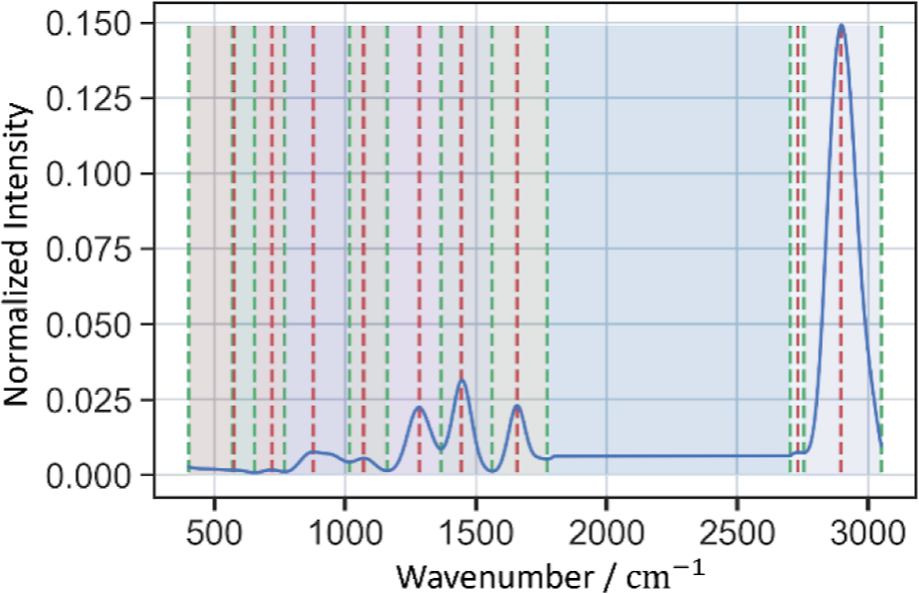
Zones limits for spectra: red lines represent peak positions, and green lines represent valleys.

Our approach initiates by defining zone boundaries based on a single spectrum or complete data set for local or global understanding, respectively. This process can be achieved using several techniques, such as detecting local extrema within the spectrum. Mathematically, a local extremum (maximum or minimum) is identified when the derivative of the function representing the spectrum, denoted as *f*(*x*), is zero (*f*′(*x*) = 0). The sign of the second derivative determines the nature of these extrema (maximum or minimum). Let us denote a set of zones *B* = {*b*_1_,*b*_2_, . . . , *b*_*n*_}, where each zone *b*_*i*_ is defined as an interval [*v*,*w*] on the spectrum, with *v* and *w* representing two different wavenumbers. Figure 2 presents the calculated zones for a spectrum, where red lines indicate peak positions and green lines represent the valleys. The zones were determined by identifying the valleys between peaks in the spectra. However, users can also define the zones based on the domain-specific knowledge.

#### 2.4.2 Group Perturbations

We construct a set of perturbed spectra denoted as *Z* = {*z*_1_,*z*_2_, . . . , *z*_*n*_}, with each sample *z*_*i*_ representing a different variation of the input spectra. Depending on the data availability or preference, these perturbed spectra are either derived from a data set or generated from the input data. We consider three alternatives for generating perturbed spectra depending on the data set’s availability and diversity. First, if the data set is comprehensive enough to cover all possible spectra variations under study, it can be directly used to substitute whole zones. For instance, a zone *b*_i_ in *x* can be replaced with its corresponding zone *b*_*i*_ from the available data, thus creating a perturbed spectra *z*_*i*_. However, it can be computationally expensive due to the large number of comparisons needed. Second, if the data set lacks sufficient diversity, it can be enhanced by applying transformations *T*, such as zone sharpening or smoothing, to introduce a broader range of variations. While this increases the diversity of the perturbed samples, its effectiveness depends on the chosen transformations, which might introduce unrealistic spectral variations that do not represent the true data distribution of the spectra.

Third, in the absence of a data set, transformations can be applied to the input data to create a set of perturbed spectra *Z*. However, the diversity of the perturbed samples may be limited by the transformations applied to a single input instance, potentially failing to capture the full range of possible variations present in a more comprehensive data set.

#### 2.4.3 Coefficient Computation

LIME scores and SHAP values are computed for groups of features instead of individual ones, as described in Sections 2.1 and 2.2. This methodology allows the assessment of collective feature impact, providing a better understanding of the model’s behavior.

### 2.5 AI Explanation Plots

We compared different strategies for explaining local predictions and found that waterfall plots, relevance coefficients, and saliency maps are highly valuable. These explanations help us understand how the features in the input data contribute to its prediction.

Waterfall plots illustrate the path from the expected model output *E*[*f*(*x*)] to the predicted model output *f*(*x*), where each row shows individual features of positive (red) or negative (blue) contributions.

The relevance coefficient plots and saliency maps display the SHAP values or LIME coefficients that explain the contribution of all features in the final prediction. A high value could indicate a positive or negative contribution, while small values indicate no relevance.

### 2.6 Data Description

The oils measured for creating the used data set for the study were purchased from local stores in the area of Jena, Germany. The data set consists of seven independent bottles of each of the following oil types: olive oil, sunflower oil, canola oil, sesame oil, peanut oil, grape seed oil, lien seed oil, and cocos oils. The Raman measurements were performed using a commercially available Bruker MultiRAM Fourier transform-Raman spectrometer using a 1064 nm Nd/YAG laser and equipped with a cryogenically cooled Ge detector. The excitation laser power was set up to 350 mW, and the spectral resolution was 4 cm^–1^. Each Raman spectrum was obtained averaging 50 scans. For each individual oil, 10 measurements were recorded.

For data analysis, the measured database was split between a training data set consisting of four different oil bottles of each type of oil and a testing data set consisting of three different oil bottles of each type of oil. As for each independent bottle, 10 measurements were performed; the training data set consisted of 40 spectra measured from 4 independent batches (bottles) per oil type, and the testing set consisted of 30 spectra measured from 3 independent batches (bottles) per oil type. Before analysis, the Raman spectra were preprocessed through several steps, including despiking, wavenumber calibration, baseline correction, and normalization. After these adjustments, the preprocessed spectra served as inputs for the classification algorithms. More detailed information can be found in the Supporting Information, Table S1.

### 2.7 Raman Spectroscopy Characterization of Oils

**Figure 3:**
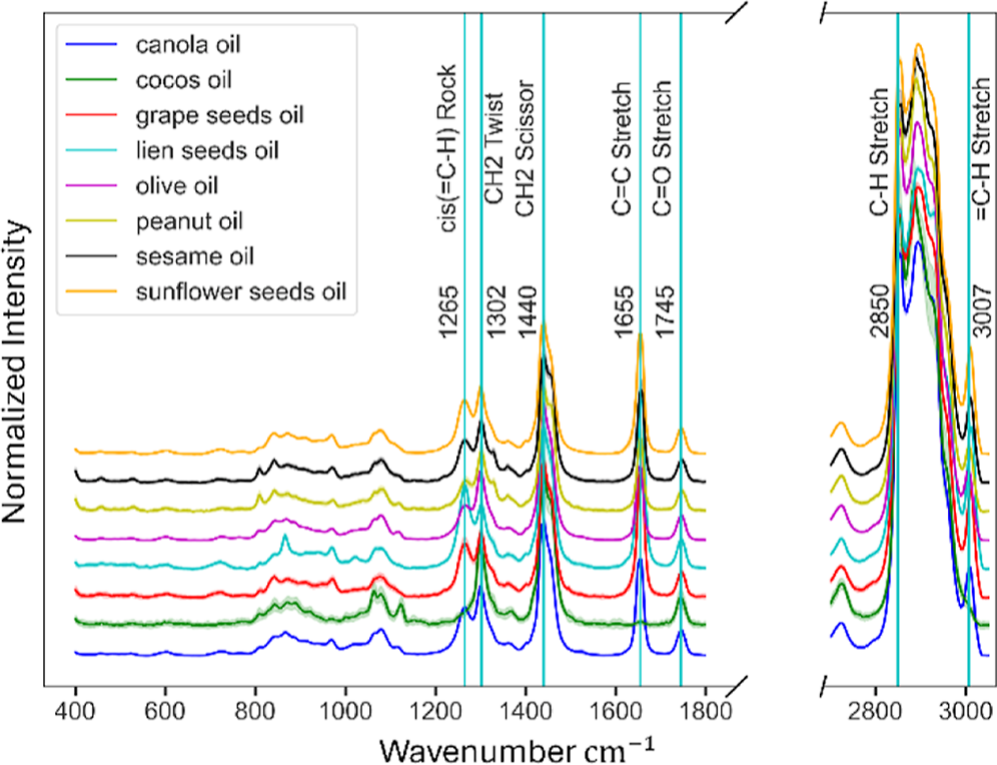
Mean normalized spectra and standard deviations of different classes after preprocessing. Blue lines emphasize wavenumber of peaks related to oils and fats.

Raman spectroscopy is an optimal technique for analyzing edible oils composed primarily of glycerol esters and fatty acids. Its strength in analyzing lipids lies in its ability to provide spectral data that exhibit distinct, well-defined peaks or regions that align directly with key molecular structures. Figure 3 and Table 1 present the distinct Raman wavenumber peaks observed across the eight analyzed oil types. The dominant signals, described as blue vertical lines, align with common fatty acid signatures. These include the peak at 760 cm^–1^ associated with C–C aliphatic stretching in lipids, the C–H bending and trans-conjugated olefinic C=C groups, approximately at 972 cm^–1^, and the CH in-plane deformation vibration found in the cis −CH=CH– segment, approximately at 1265 cm^–1^. The cis C=C bond’s stretching vibration, ν(C=C) cis, is near 1656 cm^–1^. The deformation vibrations from the saturated CH2 group, τ(CH2) and δ(CH2), situated around 1302 and 1440 cm^–1^.^29^ Oils derived from seeds rich in polysaccharides in their cell walls exhibit a pronounced peak around 1460 cm^–1^, attributed to the symmetric deformation of the −O–CH3 group, δ(CH3).^30^ The stretching vibration tied to the C=O bond in esters is noticeable at around 1745 cm^–1^.^29^

**Table 1:**
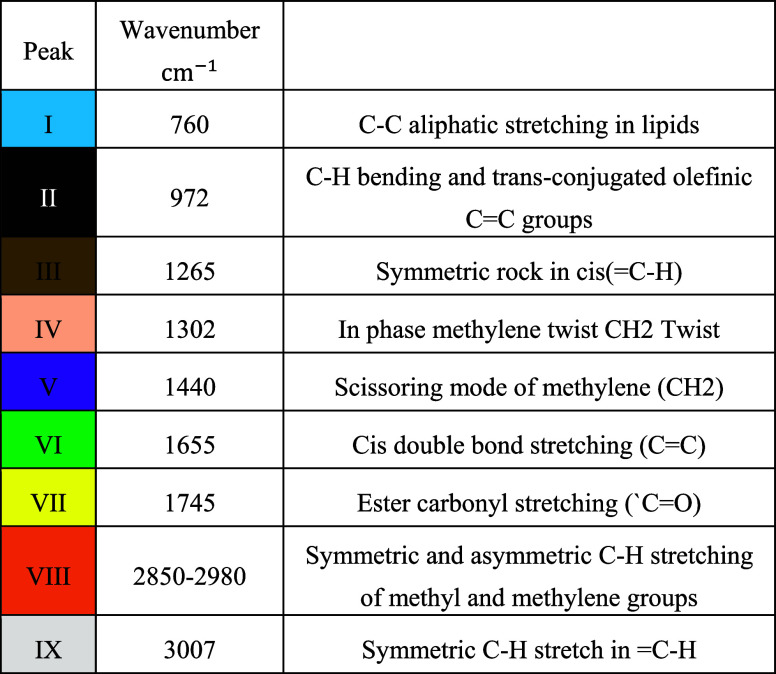
Wavenumber of Peaks Related to Oils and Fats

### 2.8 Model Description

Our primary objective was not to achieve the most accurate classification but to use a reliable and well-established deep learning (DL) model that fits our research interests and goals. Therefore, we chose a model that requires minimal parameter tuning, avoiding an exhaustive search for the optimal architecture. Consequently, we selected the RamanNet^10^ architecture, a unique approach designed to analyze Raman spectral data. RamanNet divides the input spectrum into overlapping sliding windows and processes each window through individual dense blocks, mimicking the convolution operation and enabling sparse connectivity.

This design reduces the risk of overfitting while allowing for extracting features similar to those of kernels in conventional convolutional layers. The features extracted from all of the dense blocks are concatenated. The joined features are processed by using another dense layer, dropout, and regularization. These outputs are then transformed into an embedding vector through dense layers. The model has 446,182 trainable parameters and was trained from scratch. We optimized the classification using cross-entropy loss and improved the class separation in the embedding space using triplet loss as an auxiliary loss function.

## 3 Results and Discussion

This section evaluates and interprets the RamanNet model. The evaluation focused on key performance metrics, such as precision, recall, and the F1 score, which are crucial for assessing the model’s accuracy and reliability. We used SHAP, LIME, and Zone SHAP/LIME methods to gain deeper insights into DL’s decision-making process. To enhance the transparency and reliability of the AI system, we examine and contrast the local and global explanations provided by the XAI techniques. Furthermore, we cross-check these explanations with domain knowledge to ensure the model decisions are reasonable and understandable.

### 3.1 Model Evaluation

Our Raman spectral data set consists of 320 spectra for training and 240 spectra for validation, including eight oil types. We use the provided splits to evaluate the stability of the results, as the spectra are balanced and independent. Table 2 presents the performance metrics: precision, recall, and F1 score. Recall, also known as sensitivity, shows the ability of the model to identify positive spectra correctly. Precision measures the accuracy of the positive predictions, indicating the proportion of true positive predictions out of all positive predictions made by the model. The F1 score is the harmonic mean of precision and recall, providing a single metric that balances both precision and recall. It is calculated as . The results are consistent for a network trained on a limited number of spectra that classifies eight distinct classes. Figure S1 illustrates the mean and standard deviations of spectral data across eight different classes of oils. Each class, represented by multiple batches, showcases the intraclass variations in their spectral profiles. Notably, canola oil, linseed oil, olive oil, and sunflower seed oil exhibit minimal variations between batches, indicating a higher consistency in their chemical composition. In contrast, cocos oil, grape seed oil, peanut oil, and sesame oil exhibit significant batch-to-batch variability, reflecting pronounced differences in their spectral characteristics.

**Table 2:** Precision, Recall, and F1 Score on the Eight-Oil Type Dataset for the RamanNet Model^a^

class	precision	recall	F1-score
canola oil	0.83	1.00	0.91
cocos oil	1.00	1.00	1.00
grape seeds oil	0.85	0.37	0.51
lien seeds oil	1.00	1.00	1.00
olive oil	0.83	1.00	0.91
peanut oil	1.00	0.50	0.67
sesame oil	0.80	0.67	0.73
sunflower seeds oil	0.51	0.93	0.66
mean – RamanNet	0.85	0.81	0.80
mean – PCA + LDA	0.88	0.82	0.82

a Mean metrics were for RamanNet and PCA + LDA.

Similar to previous works,^31−33^ we used principal component analysis (PCA) and linear discriminant analysis (LDA) as baselines to compare with our trained DL model. Figure S2 shows the separation of data points corresponding to different oil classes using the first two principal components (PC1 and PC2) for the training and testing data sets. PC1 accounts for 65% of the variance, while PC2 explains 12%. While the plot displays clear clustering for some oil classes, such as cocos, linseed, and olive oil, it reveals that other classes, such as sesame and peanut oils, in the testing set are dispersed and situated far from their respective cluster centers.

Figure S3 shows the cumulative explained variance versus the number of principal components. Fifteen components capture 97% of the variance, while 77 comprise 99.5%. We used 77 components for a comprehensive data representation.

Figures S4 and S5 show the variance in PC scores among different oil types and batches, providing crucial insights into the variability in spectral properties both within and between these groups. The boxplots illustrate the central tendency and spread of PC scores, with cocos oil displaying the highest variability and numerous outliers. In contrast, olive and linseed oils exhibit less variability, suggesting more consistent spectral properties. Figure S5 highlights significant variability and numerous outliers in certain batches, particularly within cocos and linseed oils. Conversely, canola and grape seed oil batches display tighter clustering around the median, indicating greater consistency.

PCA–LDA classification results for precision, recall, and F1 score are 0.88, 0.82, and 0.82, respectively. The performance of PCA + LDA is similar to that of RamanNet, indicating that our model effectively learns feature representations and is suitable for XAI methods. We aim to evaluate the performance of the trained model while acknowledging the potential for improvement through parameter tuning and other strategies, as remarked on in the model description.

### 3.2 Local Explanation

For this subsection, we have selected spectra from the class of sesame oil that was classified correctly and has an expected model output value (*E*[*f*(*x*)]) of 0.106 and a spectra prediction value (*f*(*x*)) of 0.915.

#### 3.2.1 Waterfall Plots

**Figure 4:**
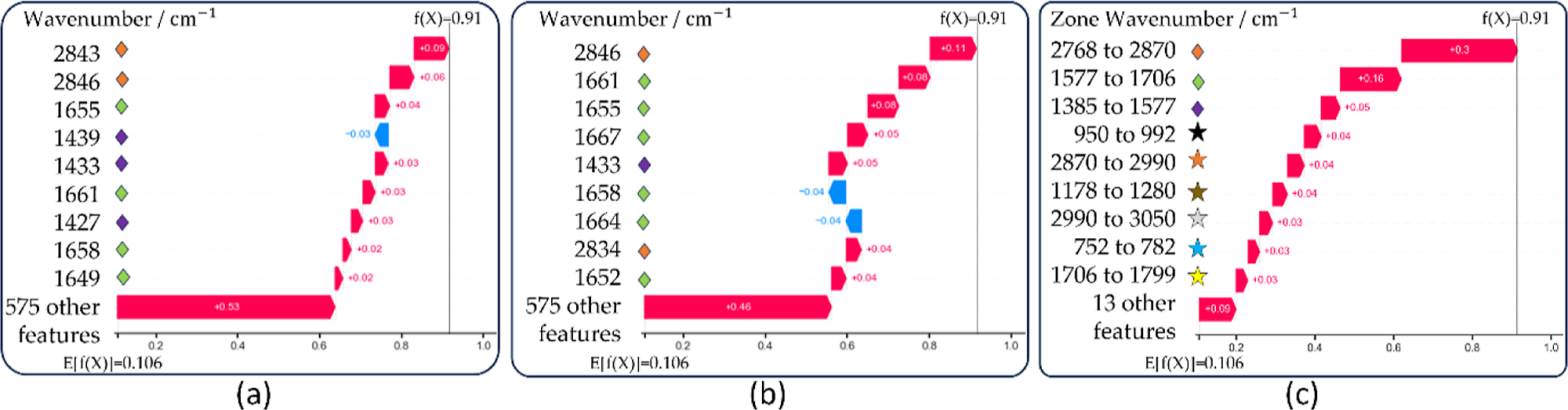
Waterfall plot for sesame oil spectra, visualizing the transition from expected to predicted output, and each row highlights feature contributions in red (positive) and blue (negative). (a) Deep SHAP, (b) sampling SHAP, and (c) zones-based SHAP.

Figure 4a,b highlights the top nine features that significantly contribute to the model’s prediction employing Deep SHAP and Sampling SHAP Explainer, respectively. The remaining 575 features are grouped together and have a combined influence. According to the results, the Deep SHAP method exhibits that the grouped influence contributes 0.530 out of the total value of 0.809. Similarly, the Sampling SHAP method shows a grouped contribution of 0.460 out of 0.809. These values are equivalent to 65.5 and 56.8% of the path from the expected value to the prediction for the Deep SHAP and Sampling SHAP, respectively, which indicates that the explanation provided covers only a portion of the prediction.

On the other hand, Figure 4c presents the top nine spectral zones that significantly contribute to the model’s prediction for zone-based SHAP Explainer, with only 13 remaining zones in the plot, representing 11.12% of the explanation.

#### 3.2.2 Analysis of Peaks Associated with Oils and Fats

Figure 4a,b displays the top nine influential features; these values can be categorized into three main groups, which are represented by colored diamonds located at positions 1433 cm^–1^(violet), 1661 cm^–1^(green), and 2843 cm^–1^(orange). These groups align with the peaks associated with oils and fats V, VI, and VIII, as detailed in Table 1. In Figure 4a, the influential values are positioned closely, indicating their interrelation. Figure 4b suggests that features may be grouped within the same clusters despite variations in order.

Figure 4c displays the top nine spectral zones, with the three highest zones containing the 2843, 1661, and 1433 cm^–1^ positions. These positions correspond to the outcomes of previous methods, underlining their consistent importance across various approaches. Zone SHAP offers supporting information about other relevant zones. Figure 4c illustrates the impact of extra peaks mentioned in Table 1, which are highlighted with colored stars reflecting peaks II (black), VIII (orange), III (brown), IX (gray), I (cyan), and VII (yellow).

#### 3.2.3 Relevance Coefficient Plots

**Figure 5:**
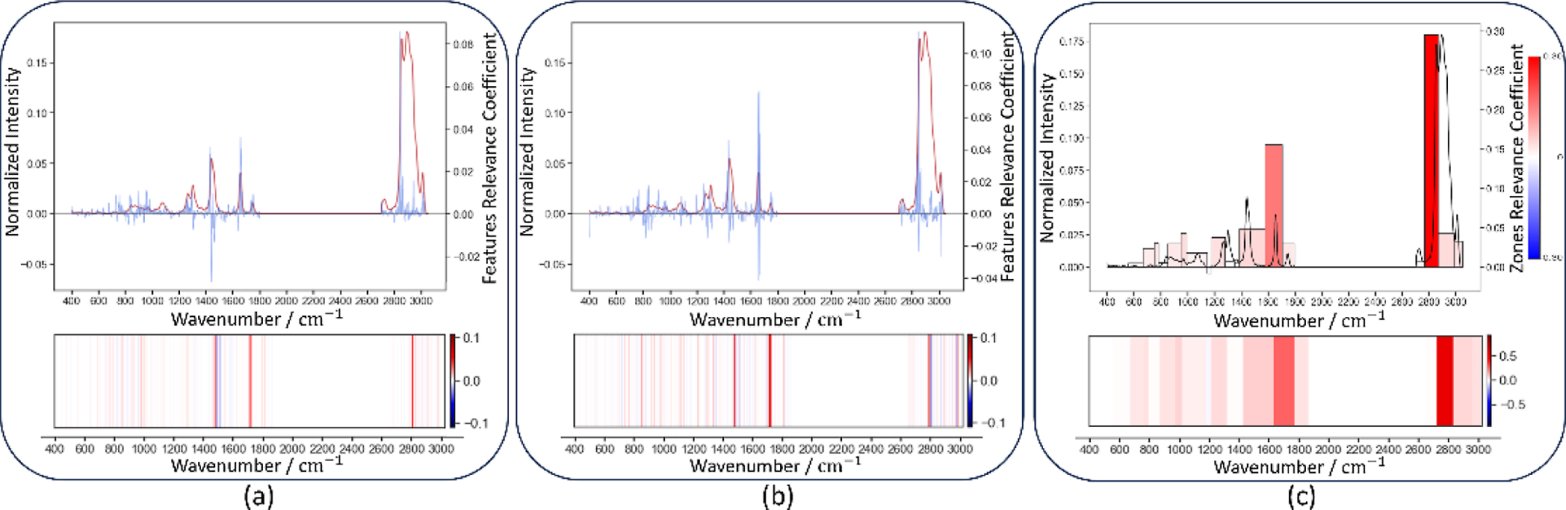
Relevance coefficient plots (top) and saliency maps (bottom) for sesame oil spectra, showing feature contribution, high values indicating strong impacts, and low values minimal relevance. (a) Deep SHAP, (b) sampling SHAP, and (c) zones-based SHAP.

Relevance Coefficient plots serve as alternative methods for visualizing the impact of all input features on model predictions. However, given the complexity and high dimensionality of our data, interpretation of these plots can be challenging. Specifically, Figure 5a,b exhibits noisy information, indicated by the fluctuating positive and negative values at neighboring wavenumbers. This variation poses challenges in interpreting SHAP values and could impact their interpretability and reliability.

Notably, the coefficient intensities are low below 700 cm^–1^. Figure 5a,b reveals a notable pattern of alternating positive and negative values across adjacent features, suggesting possible interdependencies or divergent impacts. The sequence observed at wavenumbers 1661, 1658, and 1664 cm^–1^ displays the second-highest positive and the highest and second-highest negative values, respectively. This distinct pattern highlights the importance of these features, yet their contrasting contributions complicate straightforward aggregation or interpretation. While the top features are significantly relevant, the remaining coefficients display a seemingly random distribution, complicating their interpretation. In Figure 5c, each element’s height and color denote each zone’s respective contribution to the final classification, offering a more concise and interpretable representation than seen in Figure 5a,b. This visualization provides a clearer option for comparison with domain knowledge to assess the model’s reliability. Additionally, the normalized saliency maps in Figure 5 serve as alternative representations of the SHAP coefficients. Despite there being differences in the level of noise, the strength of the intensity values, and whether the values are positive or negative, all three methods highlight the same regions. This enhances our understanding of the analytical uniformity across different approaches.

#### 3.2.4 LIME and Zones-Based LIME

**Figure 6:**
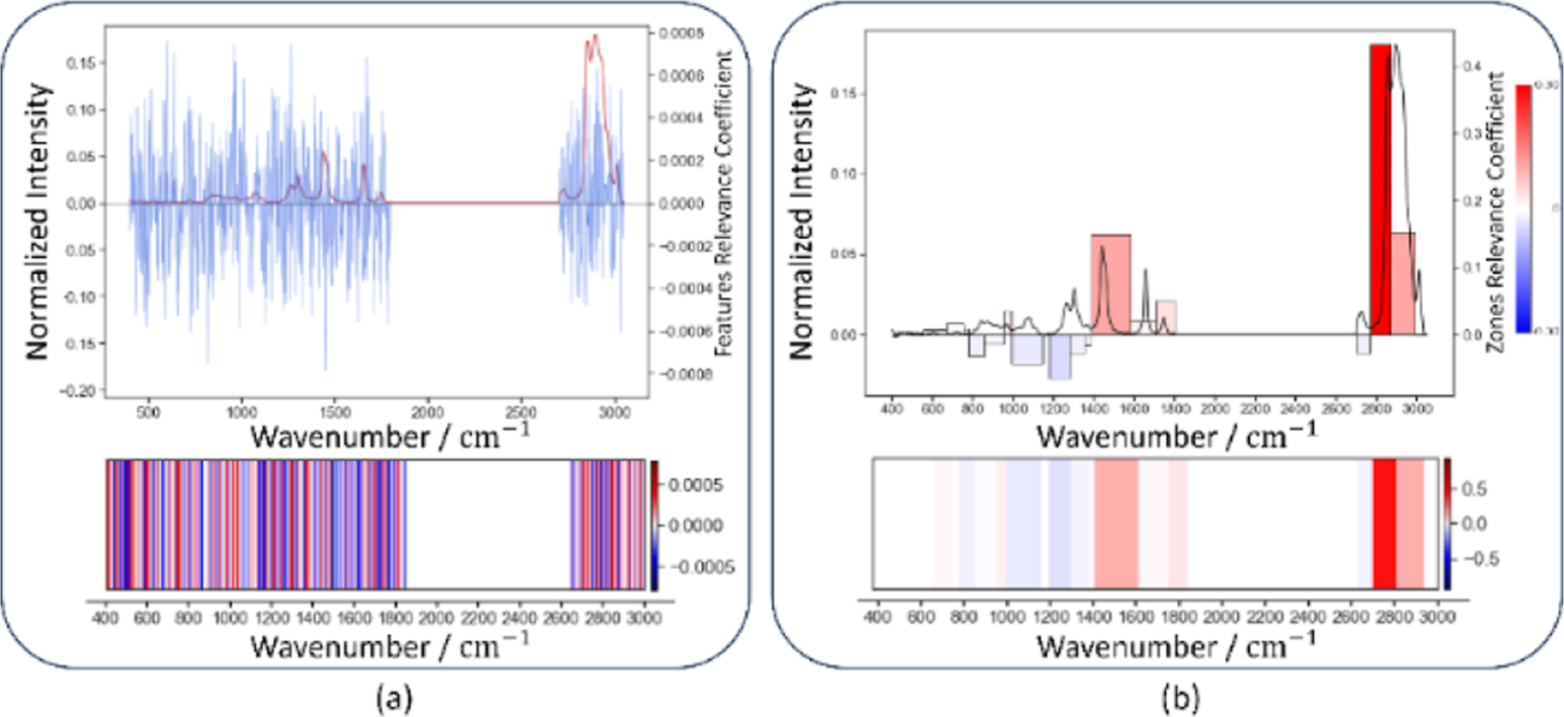
LIME coefficients for the explanation of sesame oil spectra. Top, Relevance coefficients. Bottom, Saliency maps. (a) LIME and (b) zone-based LIME.

Linear LIME lacks the additive feature attribution method, which means that the coefficients it produces cannot explain the shift from the base value (*E*[*f*(*x*)]) to the predicted outcome (*f*(*x*)). As a result, generating a waterfall plot is not possible. Figure 6 presents the relevance coefficients obtained from linear regression of LIME and zone-based LIME analysis. These coefficients provide explanations for the contributions of the features of a sesame oil spectra in the model response. The relevance coefficients are displayed at the top, while the saliency maps are at the bottom.

LIME is a technique characterized by using random perturbations, resulting in different outcomes for each iteration. Figure 6a illustrates this variability, with seemingly random coefficients indicating susceptibility to noise-induced fluctuations in the results. The depicted values are not only visually indistinct but also low in magnitude without prominent peaks. Conversely, Figure 6b demonstrates specific zones of interest. Similar to the previously discussed SHAP-based methods, our zone-based LIME approach exhibits coefficient intensities below 700 cm^–1^. Furthermore, the areas around 1661 and 1433 cm^–1^ are also part of the most relevant zones.

Although the LIME zone-based methodology produced better results than LIME, slight differences in outcomes between iterations can occur due to LIME’s inherent variability. This inconsistency highlights our preference for using SHAP as a more reliable and robust explainable AI strategy, especially in Raman spectral analysis.

### 3.3 Global Explanation

**Figure 7:**
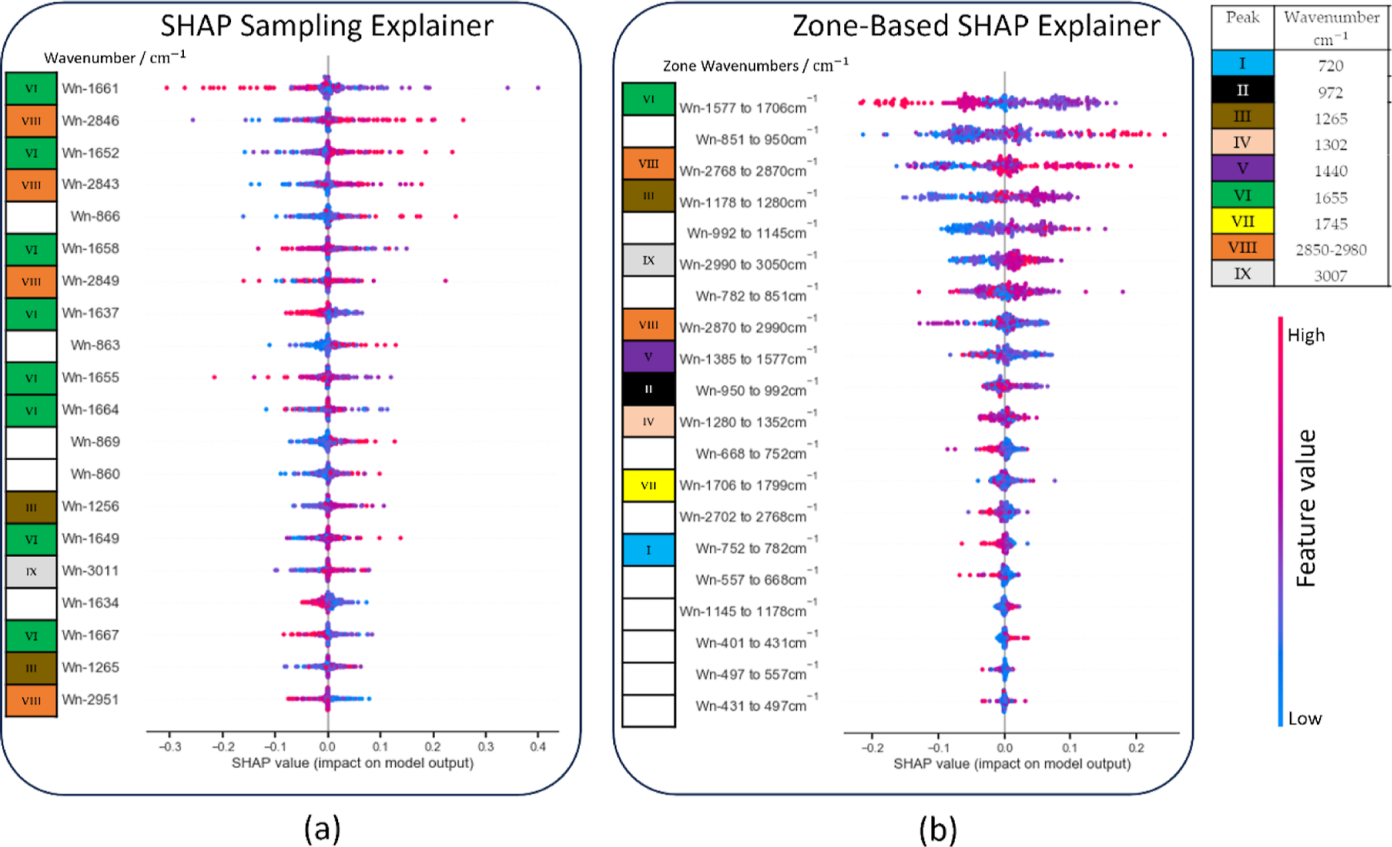
Summary plots illustrating SHAP values for the class “cocos oil” predictions for (a) SHAP sampling and (b) zones-based SHAP Explainers.

Figure 7 contains two summary plots that visualize the factors that contribute to classifying all testing spectrum into the “cocos oil” class using both the SHAP Sampling and zones-based SHAP Explainers. Each summary plot displays elements vertically in descending order of importance, with the color indicating their feature values; blue represents low intensity, while pink represents high intensity. The horizontal axis represents the magnitude of SHAP values and their impact on the classification outcome. Figure 7a illustrates the influence of the individual features. Conversely, Figure 7b shows the impact of each spectral zone. The length of the bars represents the proportions of the feature’s influence.

Additionally, in the summary plots, each row is marked with a peak number when the feature values closely match the peaks from Table 1. Rows without close correspondence remain unlabeled. The summary plots offer a comprehensive overview of the top 20 features, while the SHAP Sampling explainer displays features for four distinct peaks in Table 1. It is essential to note that Peaks I, II, IV, V, and VII do not have any corresponding features for SHAP Sampling explainer, which implies their reduced significance. Additionally, the data has some redundancy, as eight features are closely aligned with Peak VII, and four are located around Peak VIII. Despite being informative, the plot has certain limitations, such as the potential overemphasis on highly correlated features, which may overshadow other significant features. Additionally, some important peaks can be excluded due to the lack of corresponding features identified by the SHAP Sampling explainer. These limitations can lead to an incomplete representation of the model’s behavior, as not all relevant features and peaks are adequately highlighted.

Our methodology divides the features into distinct zones, providing a more precise representation of the peaks in Table 1, ranging from I to IX. This summary plot enhances clarity and aligns well with the findings from the SHAP Sampling Explainer. It highlights the significance of Peaks VII and VIII, with Peaks III and IX following in importance.

## 4 Conclusions

Our study focuses on the relationship between interpretability and accuracy in deep learning models used for 1D spectral analysis. We have found that while methods like SHAP and LIME are commonly utilized for explainable AI; they face particular challenges when dealing with complex spectral data. These methods struggle with computational demands and often fail to produce meaningful interpretations that align with the real world, especially in spectral analysis, where reliance on individual feature perturbations can lead to unrealistic interpretations.

To address these issues, we have developed an innovative approach that focuses on “spectral zones” instead of isolated features. This approach allows for a more acceptable and physically grounded interpretation of the spectral data. By adapting LIME and SHAP to adjust group perturbations, our method is able to achieve a deeper and more comprehensive understanding of the model’s behavior. Furthermore, the inclusion of domain-specific knowledge to define these spectral zones could enhance the adaptability of our method to different contexts and improve the accuracy and relevance of the analysis by focusing on critical spectral regions associated with specific molecular structures relevant to the context. While we have identified zones that correspond to characteristic peaks in oils, users have the flexibility to designate zones that align with their unique domain insights or analytical preferences. This adaptability ensures the relevance of our method across a variety of applications, providing a tailored and efficient way to delineate the spectral zones.

We recommend the use of user-defined transformations to generate a diverse and meaningful set of perturbations, allowing for a thorough exploration of the spectral data variations. Moreover, our approach emphasizes the importance of analyzing the full spectrum of data, moving beyond a focus on predominant features to capture a more comprehensive view of the model’s predictions, which is particularly beneficial in complex settings with nonlinear feature interactions.

We found that the main features used by the trained model for the spectral analysis of oils and fats can be classified into three main groups. These groups align peaks related to oils and fats, which verifies that our model uses adequate information and has no bias. Our zone SHAP approach not only confirms these clusters but also shows other spectral zones, expanding our scope of analysis. This highlights the ability of our method to ignore redundancy and provide a detailed, reliable, and clearer identification.

Finally, based on our observations, we advise against using LIME for Raman spectral analysis due to its inconsistency and variability. Instead, we recommend the zone-based SHAP approach as a more stable and reliable alternative for explainable AI in this context.

## Supporting Information Available

Mean and standard deviation of spectral data for batches across eight different classes of oils, principal component analysis showing the first two principal components for the training and testing datasets, cumulative explained variance as a function of the number of principal components, variance in principal component scores among different oil types and batches, weighted F1 score as a function of the number of principal components for training and validation data, comparison of cumulative sum of SHAP values ordered by relevance using two methods - zone-based SHAP and deep SHAP, waterfall plots, relevance coefficient plots, and oils measured (PDF)
