# Supplementary material for: Spectral Zones-Based SHAP/LIME: Enhancing Interpretability in Spectral Deep Learning Models Through Grouped Feature Analysis
Source: Anal Chem. 2024 Sep 18;96(39):15588–97. doi: 10.1021/acs.analchem.4c02329 (PMC11447665; doi:10.1021/acs.analchem.4c02329)
Supplement: Supplementary file 1 — ac4c02329_si_001.pdf [file ac4c02329_si_001.pdf]

Supporting material

Spectral zones based SHAP / LIME: Enhancing Interpretability in Spectral Deep Learning Models through Grouped Feature Analysis.

Jhonatan Contreras[1,2], Andreea Winterfeld[1,2], Juergen Popp[1,2] and Thomas Bocklitz[1,2*]

1 Institute of Physical Chemistry (IPC) and Abbe Center of Photonics (ACP), Friedrich Schiller University Jena, Member of the Leibniz Centre for Photonics in Infection Research (LPI), Helmholtzweg 4, 07743 Jena, Germany.

2 Leibniz Institute of Photonic Technology, Member of Leibniz Health Technologies, Member of the Leibniz. Centre for Photonics in Infection Research (LPI), Albert Einstein Straße 9, 07745 Jena, Germany.

*Correspondence: thomas.bocklitz@uni-jena.de

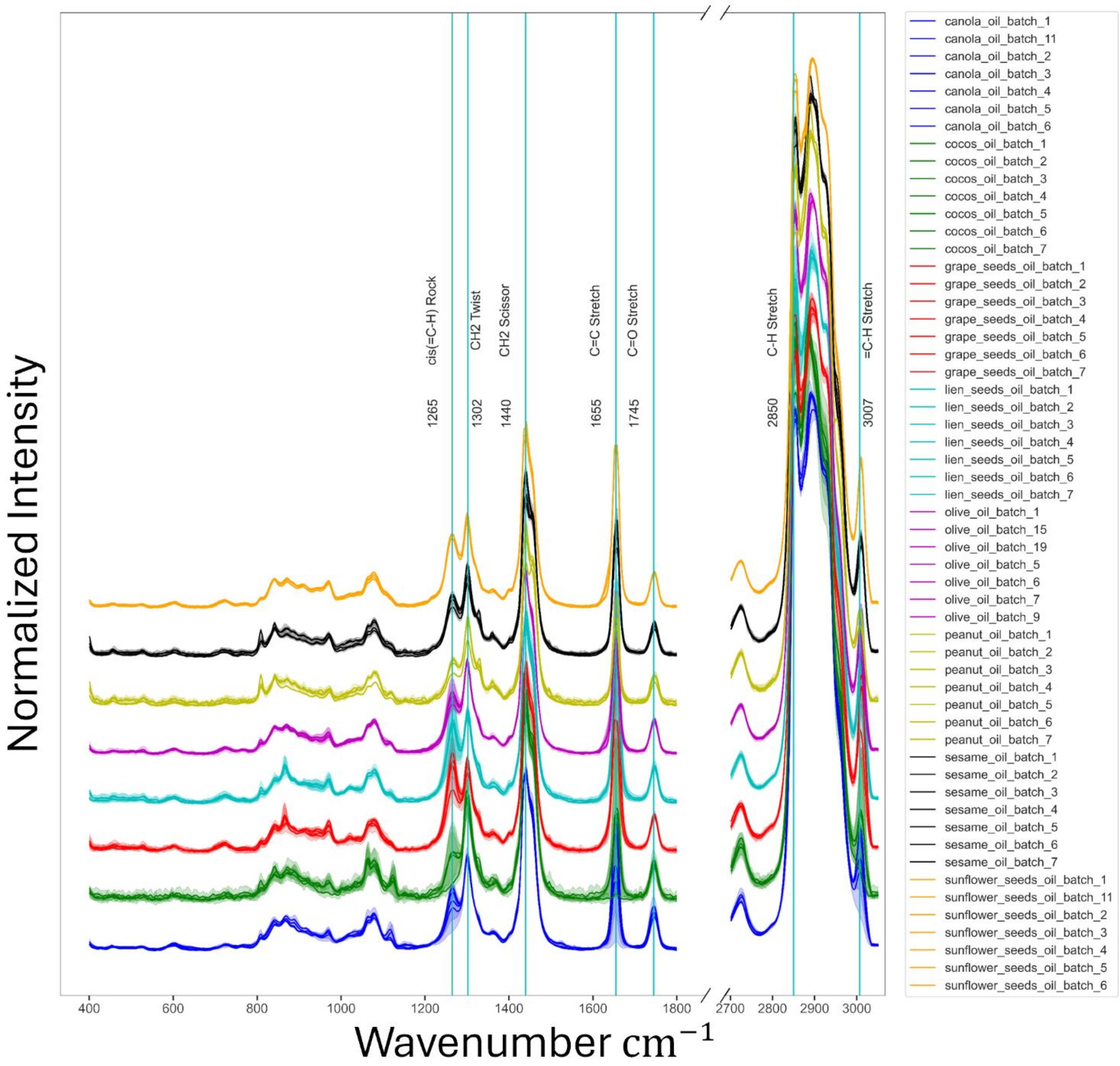

Figure S 1 Mean and standard deviation of spectral data for batches across eight different classes of oils. Each spectrum represents multiple batches within each oil class, illustrating intra-class variations. Canola oil, linseed oil, olive oil, and sunflower seed oil show minimal variations between batches, indicating consistent chemical composition. Conversely, coconut oil, grape seed oil, peanut oil, and sesame oil exhibit greater variability between batches, reflecting more pronounced differences in their spectral characteristics. Blue lines emphasize wavenumber of peaks related to oils and fats.

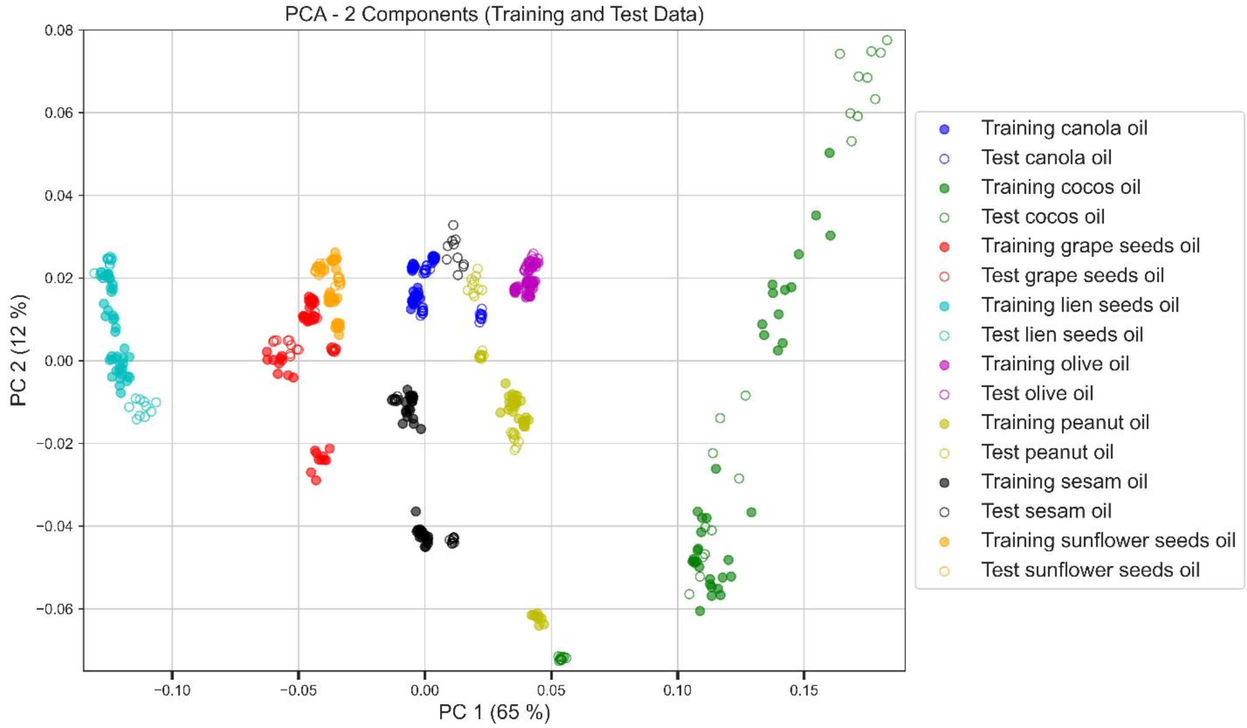

Figure S 2 Principal Component Analysis (PCA) showing the first two principal components (PC1 and PC2) for the training and testing datasets. PC1 explains 65% of the variance, while PC2 accounts for 12%. The plot illustrates the separation of different oil classes.

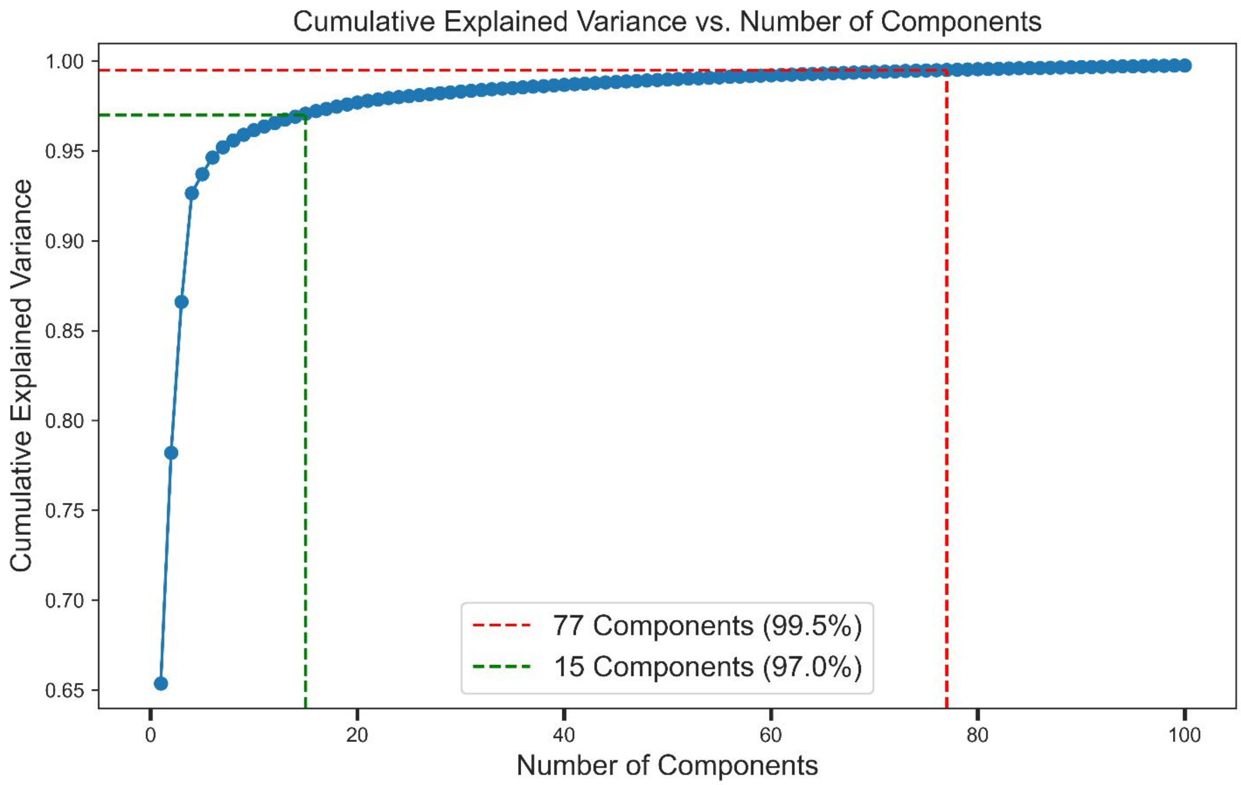

Figure S 3 Cumulative explained variance as a function of the number of principal components. 15 principal components account for 97% of the total variance, while 77 components capture 99.5% of the variance.

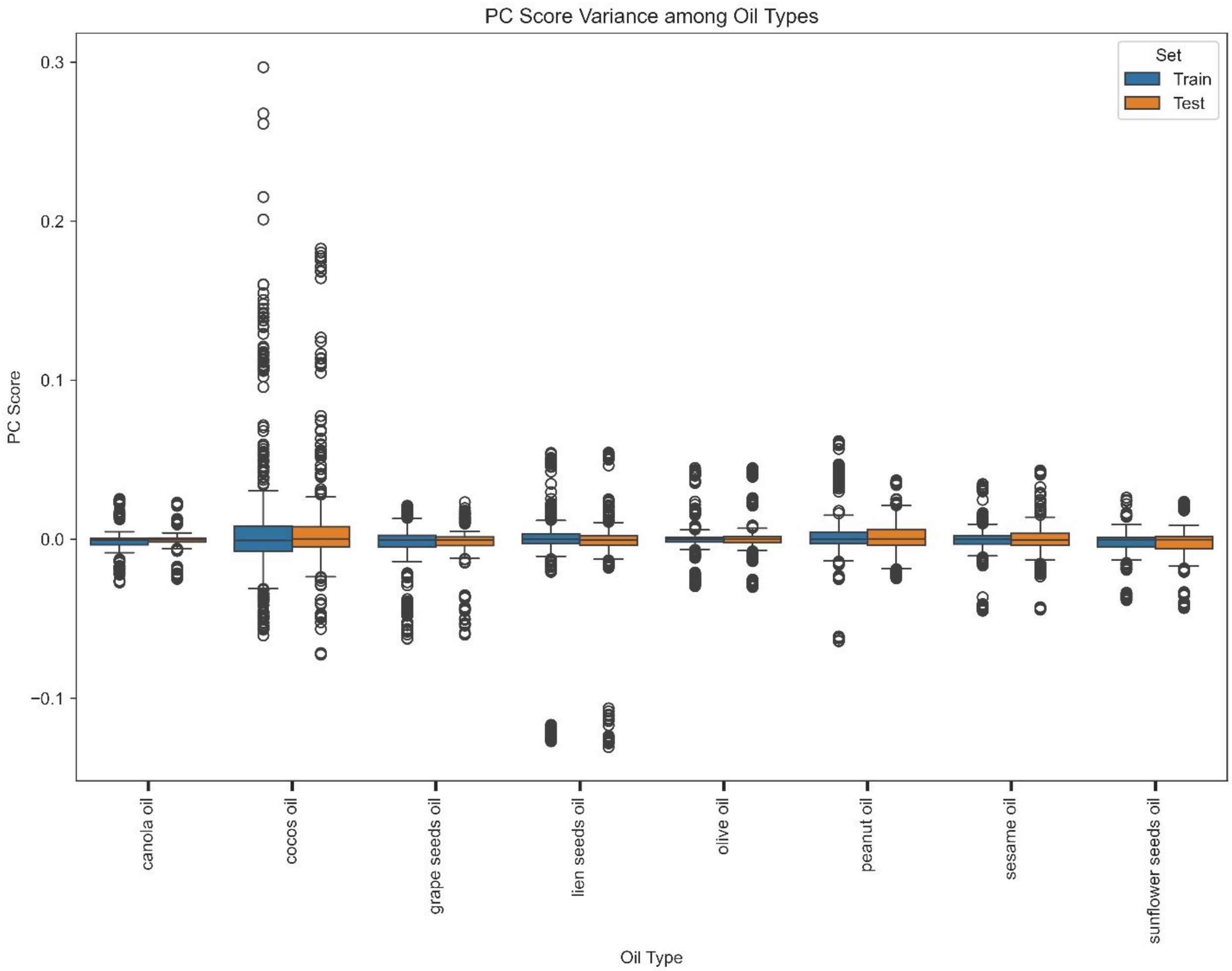

Figure S 4 Variance in principal component (PC) scores among different oil types. The boxplots illustrate the central tendency and spread of PC scores, with coconut oil displaying the highest variability and several outliers, indicating a broad range of spectral characteristics. Olive and linseed oils exhibit less variability, suggesting more consistent spectral properties.

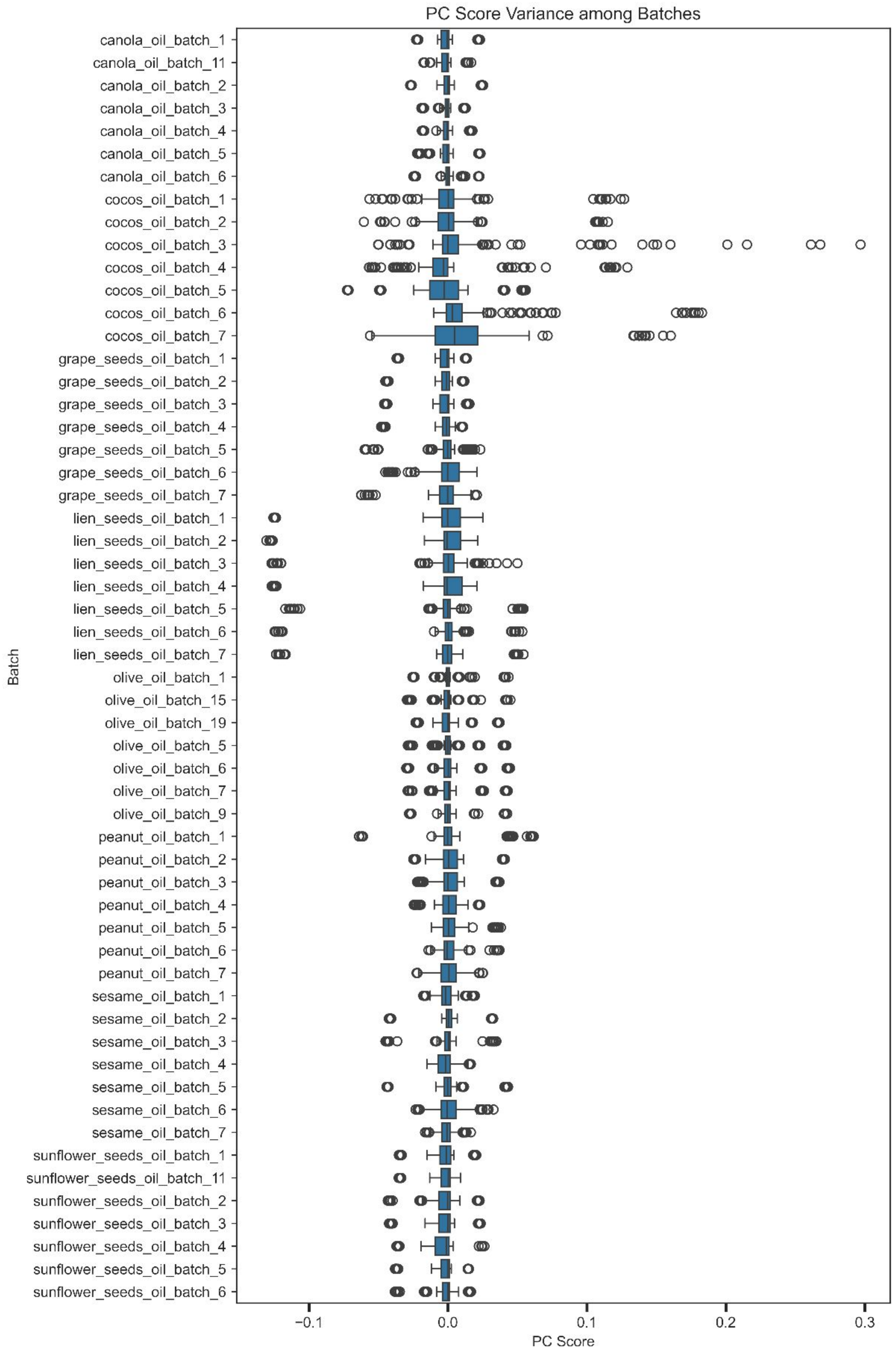

Figure S 5 Variance in principal component (PC) scores among different oil batches. The boxplots highlight significant variability and numerous outliers in certain batches, particularly within coconut and linseed oils. Conversely, canola and grape seed oil batches display tighter clustering around the median, indicating greater consistency.

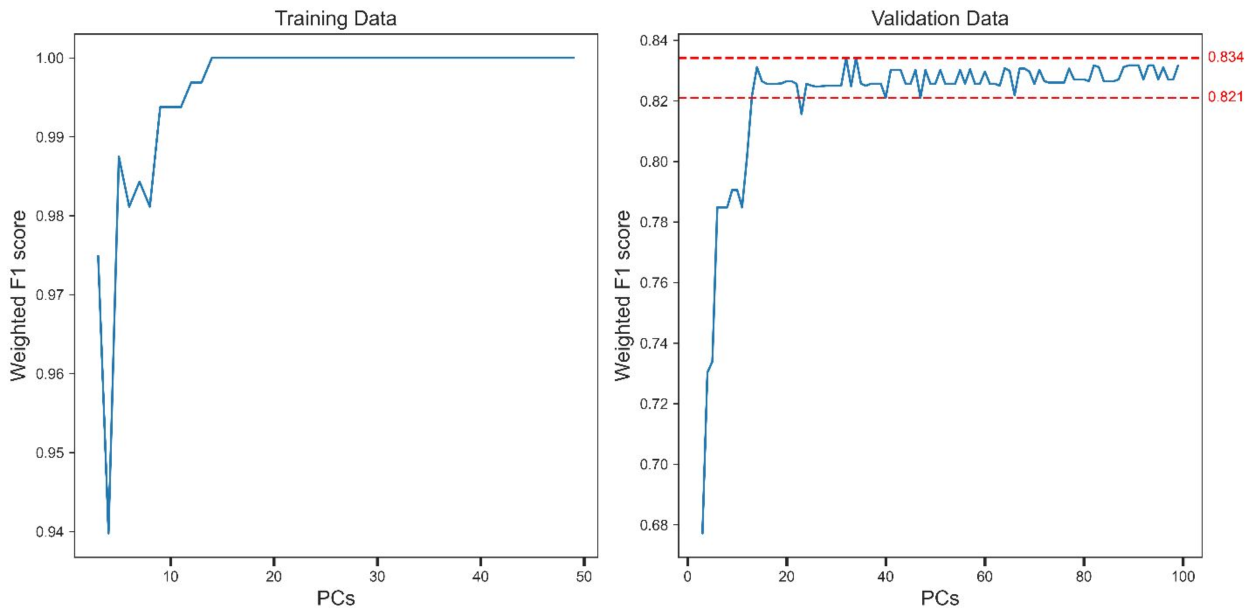

Figure S 6 Weighted F1 score as a function of the number of principal components (PCs) for training and validation data. (left) training data, indicating near-perfect classification performance as the number of PCs increases. (right) validation data, highlighting the stabilization of the score around 0.821 to 0.834 for PCs greater than 20. This suggests that while additional PCs capture more variance, their contribution to improving model performance beyond 20 PCs is marginal for the validation set.

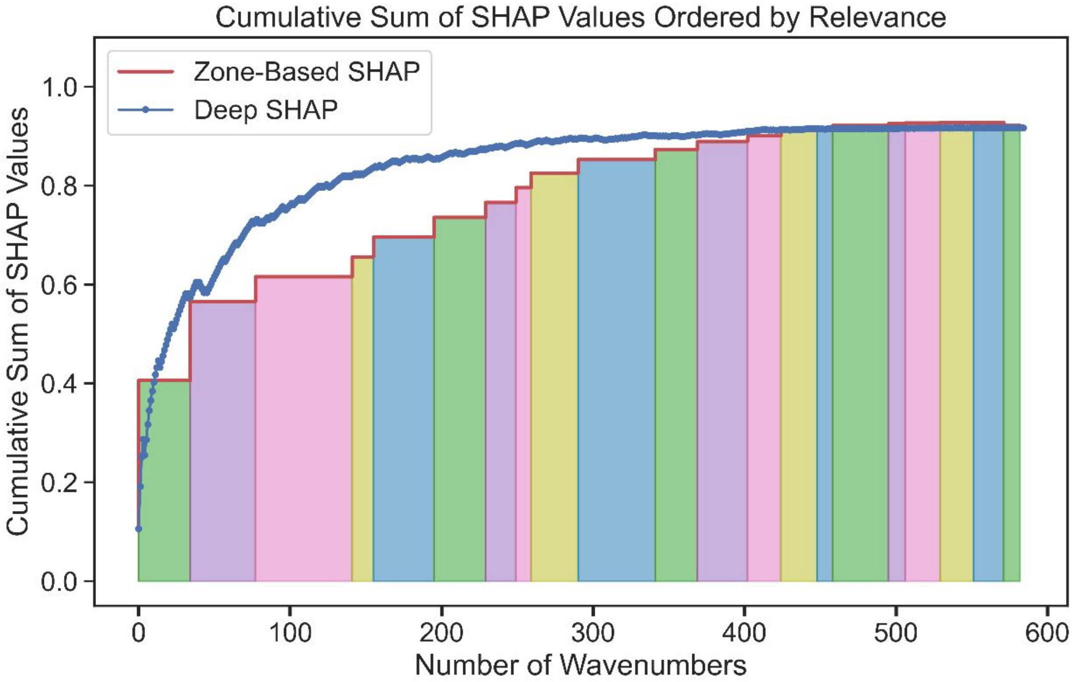

Figure S 7 Comparison of Cumulative Sum of SHAP Values Ordered by Relevance using two methods - Zone-Based SHAP and Deep SHAP. The Deep SHAP is represented by a blue line, while the Zone-Based SHAP is represented with filled step bars. The top nine zones contain 34, 43, 64, 14, 40, 34, 20, 10, and 31 wavenumbers, respectively. The top 9 zones (with >88% explainability) include 290 wavenumbers, covering approximately 49.6% of the original features. In contrast, the bottom 13 zones, comprising 294 wavenumbers, account for about 50.3% of the features but explain only 11% of the model's predictions.

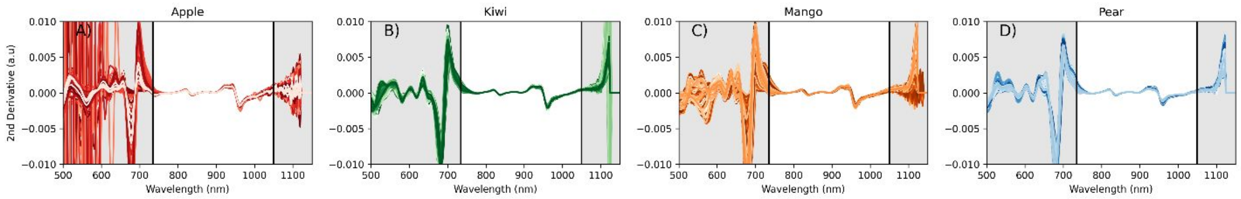

Figure S 8 Multifruit dataset. "Deep Tutti Frutti: Exploring CNN Architectures for Dry Matter Prediction in Fruit from Multi-Fruit Near-Infrared Spectra" by D. Passos and P. Mishra, published in Chemometrics and Intelligent Laboratory Systems (2023). The spectral data was pre-processed to the 2nd derivative. The target variable (Y data) corresponds to the Dry Matter content for each sample.

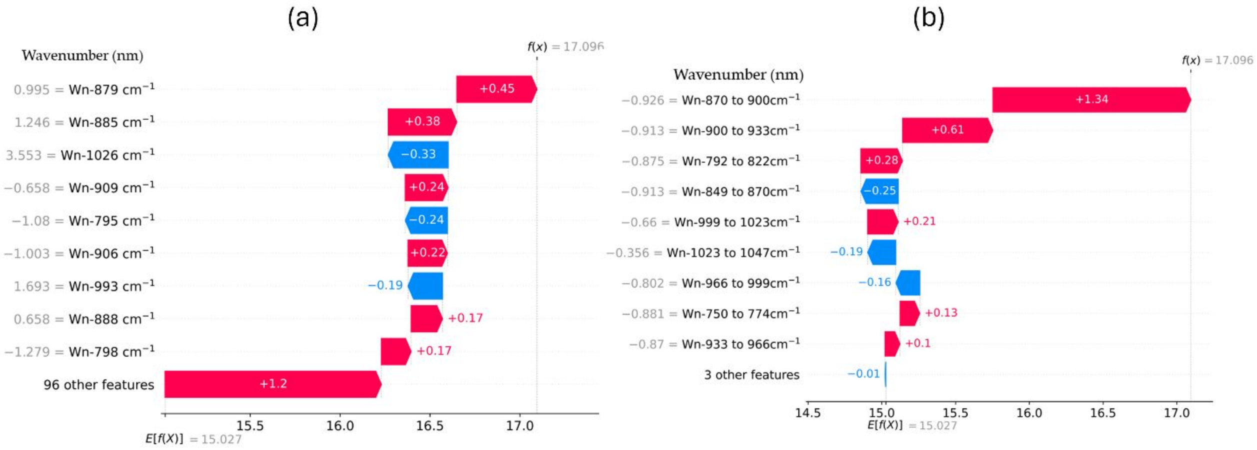

Figure S 9 Waterfall plot for a spectrum (Multifruit dataset), visualizing the transition from expected to predicted output, each row highlights feature contributions in red (positive) and blue (negative). (a) Sampling SHAP. (b) Zones-Based SHAP.

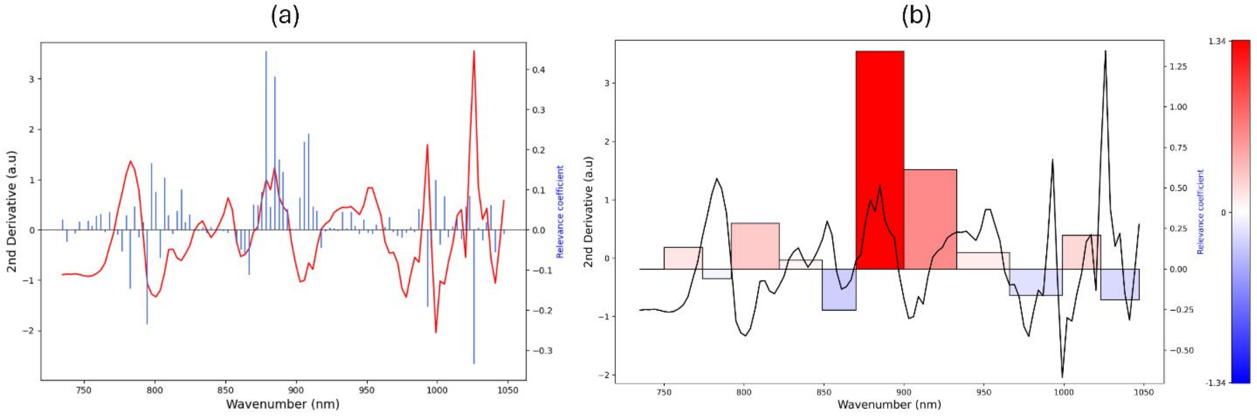

Figure S 10 Relevance coefficient plots a sample spectrum (Multifruit dataset), showing feature contribution, high values indicating strong impacts and low values minimal relevance. (a) Sampling SHAP, (b) Zones-Based SHAP.

Table S1 Supporting information oils measured

Type	Producer	Lot	Product code	Date of bottling	Country of origin	Used for
sesam oil	Alnatura	18900261	4 104420 204508	17.09.2021	Germany	Training
sesam oil	Bamboo Garden	LC00301	4 023900 261612	30.08.21	Germany	Training
sesam oil	Bio Zentrale	L 3 A 092	4 005009 103017	30.07.21	Germany	Training
sesam oil	Pagra natur	De-Ökö-006	X000E9F0YV	-	Germany	Training
peanut oil	Kunella Feinkost	L12203	4 400050	02.04.76	Germany	Training
peanut oil	Asia Green Garden	-	29008263	28.02.19	Germany	Training
peanut oil	Kunella Feinkost	L4403	4 400050 320046	01/12/21	Germany	Training
peanut oil	Kunella Feinkost	L5102	4 400050 320046	02/01/22	Germany	Training
olive oil	Denocciolato natives	-	4 044628 907941	02.01.2021	Italy	Training
olive oil	Rewe Bio	RW1301120A	4 388844 267336	10.05.21	Greece	Training
olive oil	La Espanola	L B4N090919	8 410660 511709	09.11.20	Spain	Training
olive oil	Alnatura	65190164	4 104420 031050	12.04.21	Germany	Training
canola oil	Buttella	36162	-	12.11.20	Germany	Training
canola oil	Teutoburger Ölmühle	36964	4 260014 350508	15.05.21	Germany	Training
canola oil	Jeden Tag	-	4 306188 049128	10.05.21	Germany	Training
canola oil	Rapso	L20057	9 001466 207911	28.02.21	Germany	Training
grape seeds oil	Premium Nature	X001033IL5	8 57646 00617 6	30.12.21	USA	Training
grape seeds oil	Brändle	616	4 008722 008119	-	Germany	Training
grape seeds oil	Kunella Feinkos	L2205	4 00050 320305	01.07.21	Germany	Training
grape seeds oil	Estyria Naturprodukte GmbH	L1270133001	9 009770 102434	05.11.21	Germany	Training
sunflower seeds oil	Thomy	143818427/100143829	4 005500 087151	30.11.18	Germany	Training
sunflower seeds oil	Alnatura	65440184	4 104420 031104	02.07.21	Germany	Training
sunflower seeds oil	Jeden Tag	-	4 306188 049135	12.03.21	Germany	Training
sunflower seeds oil	Kunella Feinkost	L3405	4 400050 320152	30.10.20	Germany	Training
lien seeds oil	Erfurter Ölmühle	49002V11	4 048097 951214	02.07.19	Germany	Training
lien seeds oil	Erfurter Ölmühle	49002V11	4 048097 951214	18.04.18	Germany	Training
lien seeds oil	Kunella Feinkos	L0104	4 400050 321005	10/05/21	Germany	Training
lien seeds oil	Demeter	CH 925	4 045178 002643	12/08/21	Germany	Training
cocos oil	Alnatura	45109 V4	4 104420 180833	31.01.22	Germany	Training
cocos oil	Alnatura	45111V2	4 104420 180833	30.11.21	Germany	Training

cocos oil	Dr Schlimcho	-	4 000400 002006	-	Germany	Training
cocos oil	cocos	20004241	4 260583 840134	09/08/22	Germany	Training
sesam oil	Brändle	-	4 008722 016114	-	Germany	Testing
sesam oil	Kunella Feinkos	L5104	4 400050 320053	02.01.2022	Germany	Testing
sesam oil	vom Fass	07/16 – 00059	4 044628 800594	02.01.2021	-	Testing
peanut oil	Ölmühle Solling	20/00771	4 031192 151115	20/01/22	Germany	Testing
peanut oil	Kunella Feinkost	L21203	4 400050 320046	30.09.18	Germany	Testing
peanut oil	Fleur	L21203	4 260304 332603	-	Germany	Testing
olive oil	Chania Kritis	L9180A XA-1722905/19	4 316268 583145	05.10.20	Greece	Testing
olive oil	EDEKA	LE34-02	4 311596 421626	30.02.2020	Germany	Testing
olive oil	Rewe	LH29-2 116020 XA-0623692/20	4 388860 662108	25.02.21	Greece	Testing
canola oil	Ja Rewe	680 1447 88440	4 388844 243439	-	Germany	Testing
canola oil	Kaufland	-	-	-	Germany	Testing
canola oil	Teutoburger Ölmühle	37060	4 260014 351550	21.05.21	Germany	Testing
grape seeds oil	Brändle	344	4 008722 008119	30.06.21	Germany	Testing
grape seeds oil	Kunella Feinkost	L3604	4 400050 320305	01.10.18	Germany	Testing
grape seeds oil	Kunella Feinkos	L2502	4 00050 320305	15.07.21	Germany	Testing
sunflower seeds oil	Netto Marken-Discount	-	4 316268 427548	-	Germany	Testing
sunflower seeds oil	EDEKA	-	4 311596 413256	-	Germany	Testing
sunflower seeds oil	Thomy	L01580759	4 005500 087151	30.09.21	Germany	Testing
cocos oil	Premium Nature	-	8 57646 00618 3	30.01.22	USA	Testing
cocos oil	cocos	20000741	4 260175 670798	22.09.21	Germany	Testing
cocos oil	Ölmühle Solling	20/00070	4 031192 182195	17.01.22	Germany	Testing
lien seeds oil	Alnatura	L1 100001 4924	4 104420 025929	19/11/21	Germany	Testing
lien seeds oil	Bio Zentrale	L 5 A 186 21:57	4 005009 102997	30.07.20	Germany	Testing
lien seeds oil	GutBio	L7	2903 4079	30.09.18	Germany	Testing
